# A systematic review of silymarin and silibinin mechanisms for attenuating cerebral ischemia-reperfusion injuries

**DOI:** 10.22038/ajp.2024.25370

**Published:** 2025

**Authors:** Hossein Mardani-Nafchi, Saeid Heidari-Soureshjani, Sahar Rostamian

**Affiliations:** 1 Ph.D. Student of Pharmacology, Shahid Beheshti University of Medical Sciences, Tehran, Iran; 2 Modeling in Health Research Center, Shahrekord University of Medical Sciences, Shahrekord, Iran; 3 Harvard Medical School, Department of Medicine, USA

**Keywords:** Silymarin, Carsil, Silybin, Ischemia-reperfusion injury, Reperfusion injury

## Abstract

**Objective:**

Cerebral ischemia-reperfusion injury (CI/RI) can lead to a range of impairments and even permanent disability.This systematic review was designed to comprehensively investigate the biological effects of silymarin and silibinin in mitigating CI/RI.

**Materials and Methods:**

To find studies published before January 02, 2024, a comprehensive electronic search was carried out across multiple databases, including Cochrane Library, PubMed, Embase, Web of Science, and Scopus. Data including study characteristics, methods, and biological mechanisms were extracted.

**Results:**

Silymarin and silibinin potentially improved endogenous antioxidants and reduced lipid peroxidation, nitric oxide (NO), and malondialdehyde (MDA) levels. They also enhances the nuclear factor erythroid 2-related factor 2 (Nrf2) expression and upregulated HO-1 and NAD(P)H: quinone oxidoreductase 1 (NQO1). They also protected the activity of Na^+^–K^+^ ATPase, activating mitochondrial membrane potential that suppresses mitochondrial permeability transition pores (mPTP). Moreover, they upregulated proliferator-activated receptor gamma coactivator 1-alpha (PGC1-α), uncoupling protein 2 (UCP2), nuclear respiratory factor 1 (NRF1), and reduced inducible-NO synthase (iNOS), cyclooxygenase-2 (COX-2), and myeloperoxidase (MPO) expression. They inhibited transcription factors, including nuclear factor-kappa B (NF-κB) and IκB-α degradation. They also attenuated tumor necrosis factor-α (TNF-α), interleukin-1β (IL-1β) and IL-6. Anti-apoptotic properties were revealed by increasing protein Bcl-2 and reducing p53, Bax, caspase-3, and 9 expressions. silymarin improves pathological changes, behavioral tests, and decreases cerebral infarct size.

**Conclusion:**

Silymarin and silibinin indicated promising effects on CI/RI through various mechanisms. However, well-designed clinical trials are needed to validate these findings in human subjects.

## Introduction

**Figure 1:**
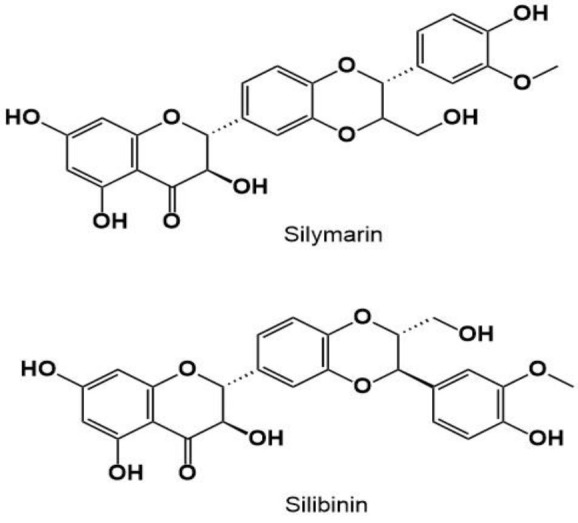
The chemical structure of silymarin and silibinin.

Cerebral ischemia-reperfusion injury (CI/RI), known as impaired blood flow to the brain, disrupts the physiological and biochemical requirements of the central nervous system (CNS) and can result in death or permanent disability (Muley et al. 2013; Zhou et al. 2022). However, unblocking the vessels can restore cerebral blood flow, and worsen brain damage known as ischemia/reperfusion (I/R) injury (Zhou et al. 2022). Various mechanisms are involved in CI/RI and they include excitotoxicity, oxidative stress, infiltration of leukocytes, mitochondrial dysfunction, complement activation, platelets aggregation and over-activation, and disturbance of the blood-brain-barrier (BBB), which consequently leads to neuroinflammation in the brain, hemorrhagic transformations, thrombosis or embolism, and irreversible neural damage cells. Currently, no treatments are available to target reperfusion injury specifically (Wang et al. 2016). While reperfusion is necessary for tissue recovery, it can aggravate injury by inducing oxidative stress, inflammation, and necrosis. Thus, it is imperative to investigate various strategies to mitigate I/R injury to optimize clinical outcomes (Orellana-Urzúa et al. 2023). One of natural substances that has revealed potent antioxidant, anti-inflammatory, and neuroprotective properties in various studies is silymarin (Aboelwafa et al. 2020; Emadi et al. 2022; Ranjan and Gautam 2023; Surai et al. 2024; Ullah and Khan 2018). Silymarin or silybin is a mixture of flavonolignans with a C_25_H_22_O_10_ molecular formula and 482.4 g/mol molecular weight that is found in *Lycium chinense*, *Silybum marianum*, and *Saururus chinensis* (Alidoost et al. 2006) (Figure 1). 

There is yet to be an ideal clinical treatment for CI/RI due to the various mechanisms involved in developing it. These complex mechanisms cause irreversible brain damage via a cascade of devastating reactions (Orellana-Urzúa et al. 2020).

Considering the high prevalence of CI/RI and ischemic stroke and its associated complications and mortality, it is crucial to comprehend the mechanisms of silymarin effects on CI/RI to develop more effective therapies.

## Materials and Methods

### Database selection, and searching strategy

We systematically searched on January 02, 2024 for literature searches in various databases including Web of Science, PubMed/MEDLINE, Scopus, Embase, and Cochrane Library, to find relevant studies. We used MeSH (Medical Subject Headings) and frequently used keywords from previous articles to enhance search precision. The keywords used were: ((“silymarin” OR “silimarin” OR “silybin” OR “silibinin” OR “milk thistle” OR “karsil” OR “carsil”) AND (“ischemia-reperfusion injury” OR “ischemia reperfusion injury” OR “cerebral ischemia-reperfusion injury” OR “cerebral ischemia reperfusion injury” OR “cerebral ischemia/reperfusion injury” OR “CI/RI” OR “cerebral hypoxia/reoxygenation injury”)). In this study, we enhanced our search strategy by incorporating relevant articles and previous review papers. We continuously refined the search process until all pertinent publications were identified and screened for inclusion in the review. Peer-reviewed articles were imported into EndNote 21.0.1 (Thomson Reuters, 25 July 2023) to detect and eliminate duplicate entries.

### Selection criteria

#### Inclusion criteria

The inclusion criteria were established according to the PICOS framework: P (participant: patients/animals/cells with CI/RI or inducing CI/RI), I (intervention: silymarin administration), C (comparison: other chemical drugs or control group), O (Outcome: efficacy of silymarin to attenuate complications due to CI/RI), S (study design: Preclinical and clinical studies), were considered inclusion criteria in this systematic review.

#### Exclusion criteria

Exclusion criteria were applied to screening irrelevant studies, including duplicate publications, case reports, conference abstracts, review articles, letters to the editor, and publications lacking full texts.

### Full-text assessment for screening process

Initially, two independent reviewers assessed the titles and abstracts of publications according to predefined inclusion and exclusion criteria aligned with the study’s objectives. Subsequently, full-text articles were meticulously examined to identify studies that met the inclusion criteria. Any discrepancies were resolved through discussion or consultation with a third reviewer to ensure accuracy and consistency in the selection process.

### Data collection and extraction

A standardized form was used to extract critical information based on the aims of the study, including study characteristics (Lead author's names, reference), study type, models (Population/animals/cells), silymarin intervention details (type of flavonolignans, concentration, and duration), intervention specifics, outcomes, and underlying mechanisms.

### Narrative Synthesis

In this stage, we summarized the critical characteristics of the included studies in a Table comprising a narrative summary of findings. We analyzed and synthesized the findings, noting similarities and discrepancies regarding the effects of silymarin on CI/RI and proposed mechanisms underlying its action.

### Reporting Guidelines

The preferred reporting items for systematic reviews and meta-analyses (PRISMA) 2020 guidelines were utilized to ensure precise and thorough reporting of the systematic review process, encompassing study selection, data extraction, and results synthesis.

## Results

### Search outcomes

**Figure 2:**
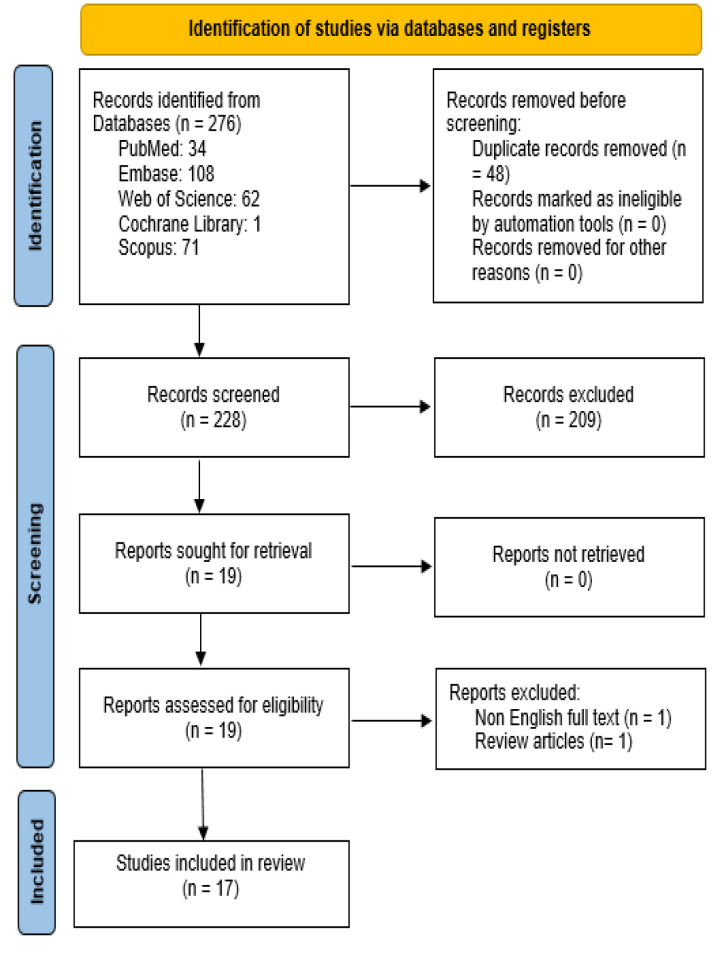
Flowchart for including studies in the systematic review

In the initial search, 276 records were included in the study, and among these titles/abstracts, 48 publications were removed due to duplication. Some articles were excluded from this systematic review due to their not fulfilling the inclusion criteria. These studies were excluded because the full text of the article was not published in English (Rui et al. 1990), as well as one study due to its design type, which was a review article (Akbari-Kordkheyli et al. 2019). Finally, 17 studies met the inclusion criteria, which we will review below. The search screening process (according to the PRISMA flowchart) is illustrated in Figure 2. 

### Description of the included studies

As seen in Table 1, silymarin and its compounds can effectively reduce the consequences of CI/RI with various antioxidant, anti-inflammatory, anti-apoptotic, and neuroprotective properties. 

**Table 1:** Characteristics of experimental studies of the effect of silymarin on CI/RI

**Lead author (Reference)**	**Silymarin or silibinin**	**Models (Animals/cells) **	**Experimental approach (Dosage and exposure time) **	**Underling mechanisms and outcomes**
Hou (Hou et al. 2010)	Silymarin or silibinin	CI/RI model in rats and microglial BV2 cells	1, 5, or 10 µg/kg (n= 5–10, for each dose) for animals and 5, 10, and 20 µM for cells, and after 24 hours, outcomes were measured.	↓ CI/RI brain infraction, protein nitrosylation, lipid peroxidation, oxidative stress, superoxide anions, iNOS, COX-2, MPO, TNF-α, -1β, and NO production. ┴ Transcription factors, including NF-κB, STAT1, and IκB-α degradation.
Raza (Raza et al. 2011)	Silymarin	Male Wistar rats with middle cerebral artery occluded	200 mg/kg body weight for 15 days	↑ GSH GPx, GR, and SOD. It also protects the activity of Na^+^–K^+^ ATPase activity. ↓ Neuronal loss and the presence of intact neurons p53, Caspase-3, and 9.
Muley (a) (Muley et al. 2012)	Silymarin	Male Wistar rats with Transient middle cerebral artery occlusion	50, 100, and 200 mg/kg, body weight, daily for 7 days.	↑ GSH, SOD, CAT, motor performance, histopathological changes, and neurological scores. ┴ Memory impairment, MDA and NO formation.
Wang (a) (Wang et al. 2012)	Silibinin	Male Sprague-Dawley rats with permanent middle cerebral artery occlusion	50 and 100 mg/kg and assessed after 24 and 72 hours	↑ Neurological scores, PAKT, pmTOR, HIF-1α, and Bcl-2. ↓ Infarct size, brain edema, Bax, NF-κB.
Muley (b) (Muley et al. 2013)	Silymarin	Wistar rats with bilateral common carotid artery occlusion	50, 100, and 200 mg/kg, b.w., p.o. daily for 7 days	↑ Behavioral, cognitive, and motor function, GSH, CAT, and SOD. ↓ Lipid peroxidation. It also improved, histopathological alterations, and cerebral infarct size.
Liu (Guo et al. 2014)	Silybin	Sprague-Dawley mice with permanent middle cerebral artery occlusion	75 mg/kg and 150 mg/kg Silybin solution per rat daily assessed after 72 hours.	↓ Brain water content of the lesion side, Bax, NF-κB protein, and mRNA. ↑ p-Akt 473, p-Akt 308, HIF-1α, and Bcl-2 protein.
Xie (Xie et al. 2014)	Silibinin	Oxygen–glucose deprivation-induced neuroblastoma SH-SY5Y cells	1–200 µM was applied for 24 hours	┴ ROS production and apoptosis by AMPK signaling activation. ↓ Caspase-3 activity and apoptosis.
Hirayama (Hirayama et al. 2016)	Silymarin	Sprague-Dawley rats with occluding bilateral carotid arteries	200 mg/kg body weight orally to the pre-and post-groups administered 12 hours to 1 week after the surgery	↓ Neuronal necrosis and autophagy.
Wang (b) (Wang et al. 2016)	Silibinin	Hypoxia/ischemia injury in the middle cerebral artery occlusion mouse model.	Mice were given 200 mg/kg (i.g.) SLB for 3 days and 0, 0.01, 0.1, 1, 10 and 100 μM for 30 min in cells	↑ Neuron viability, inhibits cell apoptosis. ↓ Procaspase-3 bal and Bcl-2, Bax, LC3-II and Beclin-1, Akt-1 and mTOR.
Chauhan (Chauhan et al. 2017)	Silibinin	Male Wistar rats with middle cerebral artery occlusion-induced focal cerebral ischemia	100 mg/kg and 200 mg/kg body weight, once daily for 7 days	↑ Neurobehavioural changes, GSH, SOD, levels. ↓ Infarct size, MDA and NO.
Gupta (Gupta and Gupta 2017)	Silymarin	Male Wistar rats with middle cerebral artery occlusion model of focal cerebral ischemia	250 mg/kg for 3 days	↑ SOD levels. ↓ MDA and NO levels.
Karabag (Karabag and Koçarslan 2020)	Silymarin	Wistar albino rats with I/R were implemented with a tourniquet	200 mg/kg Silymarin was given as ip 5 minutes before the release of the tourniquet	↓ Histopathologic damage score. ┴ Lipid hydroperoxide and total free sulfhydryl.
Moghaddam (Moghaddam et al. 2020)	Silymarin-loaded chitosan nanoparticles	Male Wistar rats with bilateral typical carotid artery occlusion	15 mg/kg orally administration started 14 days prior to CI/RI	↓ Depressive-like behaviors, infarct size, MDA, IL-6, and TNF-α. ↑ SOD, CAT, GPx, GRx, and GSH levels.
Rathore (Rathore et al. 2020)	Silymarin encapsulated inside collagen-based polymeric nanoparticles	Male albino Wistar rats with middle cerebral artery occlusion	10, 100, and 1,000 µg/kg b.wt for 7 days.	↑ Neurobehavioral, positive histopathological changes, immunohistochemical cell viability, GSH, GR, GPX, SOD, CAT, and GST. ↓ NF-κB, iNOS, and apoptotic protein Caspase-3 and infarct volume.
Mokhtari (Mokhtari Sangdehi et al. 2022)	silymarin-loaded chitosan nanoparticles	Male Wistar rats with bilateral typical carotid artery occlusion	15 mg/kg for 14 days	↓ Memory and learning impairments and Caspase-3. ↑ GSH, CAT, SOD, GPx, and GRx levels.
Pasala (Pasala et al. 2022)	Silybin phytosome	Adult Wistar rats with occlusion of bilateral common carotid arteries	20 mg/kg body weight po for 14 days	↑ GSH and SOD. ↓ TNF-α, IL‐6, cortex cell necrosis.
Li (Li et al. 2023)	Silibinin	Hypoxia/reoxygenation -treated N2a cells	5, 10, 20, 50, 100 or 200 μM for 24 hours	↑ Cell viability, GAS6/Axl expressing and SIRT1 levels. ↓ ROS generation, LDH, Bcl2/Bax and Caspase-3 ┴ Mitochondria dysfunction (↑ UCP2, NRF1, and PGC1-α), and oxidative stress (↑ Nrf2, HO-1, NQO1).

CI/RI, Cerebral ischemia–reperfusion injury; iNOS, Inducible nitric oxide synthase; COX-2, Cyclooxygenase-2; MPO, Myeloperoxidase; TNF-α, Tumor necrosis factor-alpha; IL-1β, Interleukin-1 beta; NO, Nitric oxide; NF-κB, Nuclear factor kappa B; STAT1, Signal transducer and activator of transcription 1; IκB-α, Inhibitor of kappa B-alpha; GSH, Glutathione; GPx, Glutathione peroxidase; GR, Glutathione reductase; SOD, Superoxide dismutase; CAT, Catalase; Na+–K+ ATPase, Sodium-potassium adenosine triphosphatase; MDA, Malondialdehyde; p53, Tumor protein p53; Caspase-3/9, Cysteine-aspartic proteases 3 and 9; PAKT, Phosphorylated Akt; pmTOR, Phosphorylated mammalian target of rapamycin; HIF-1α, Hypoxia-inducible factor 1-alpha; Bcl-2, B-cell lymphoma 2; Bax, Bcl-2-associated X protein; ROS, Reactive oxygen species; AMPK, AMP-activated protein kinase; LC3-II, Microtubule-associated protein 1A/1B-light chain 3 II; Beclin-1, Autophagy-related protein; Akt-1, Protein kinase B1; mTOR, Mammalian target of rapamycin; GST, Glutathione S-transferase; LDH, Lactate dehydrogenase; UCP2, Uncoupling protein 2; NRF1, Nuclear respiratory factor 1; PGC1-α, Peroxisome proliferator-activated receptor gamma coactivator 1-alpha; Nrf2, Nuclear factor erythroid 2-related factor 2; HO-1, Heme oxygenase 1; NQO1, NAD(P)H dehydrogenase quinone 1; SIRT1, Sirtuin 1; GAS6, Growth arrest-specific 6; Axl, A receptor tyrosine kinase in the TAM family.

## Discussion

### Protective role of silymarin and silibinin against CI/RI

With this pathophysiological process, treatments with antioxidant, anti-inflammatory, and anti-apoptotic effects, such as silymarin, are effective, which we will discuss in detail below.

### Antioxidant effects

Silymarin contains flavonolignans, specifically silybin, with potent antioxidant properties. These properties allow to scavenge free radicals and ROS generated during I/R, which reduces oxidative stress in the brain (Surai 2015). During I/R, excessive free radicals such as superoxide anion and hydroxyl (·OH) radicals are generated, which can lead to oxidative damage to cellular organelles and molecules including lipids, proteins, DNA and cause several complications including hemorrhagic transformation, cerebral edema, and BBB (Sun et al. 2018). Silibinin (200 µM) in Xie et al. study showed a promising effect on reducing ROS production and apoptosis by AMP-activated protein kinase (AMPK) signaling activation on oxygen–glucose deprivation-induced neuroblastoma SH-SY5Y cells (Xie et al. 2014).

Silymarin's potential to neutralize these changes has been proven to improve the activity of endogenous antioxidant enzymes, such as superoxide dismutase (SOD), catalase (CAT), and glutathione peroxidase (GPx) (Farjad and Momeni 2018). Silymarin administration (200 mg/kg/day orally) for three days can increase GSH and SOD activity. These findings indicate that silymarin may offer protective effects against oxidative damage in neural cells by inhibiting lipid peroxidation (Nencini et al. 2007). Several studies indicated that silymarin and silibinin increased GSH, GPx, GRx, glutathione reductase (GR), CAT, and SOD levels and induced anti-oxidant activity (Moghaddam et al. 2020; Mokhtari Sangdehi et al. 2022; Muley et al. 2013; Muley et al. 2012; Pasala et al. 2022; Rathore et al. 2020; Raza et al. 2011). It also reduces lipid peroxidation and MDA levels in experimental animals (Chauhan et al. 2017; Moghaddam et al. 2020; Mokhtari Sangdehi et al. 2022). 

These enzymes play a crucial role in cellular protection against oxidative stress by converting harmful ROS such as H_2_O_2_, ·OH, superoxide (O2 ^.-^), NO, and ONOO^- ^into less reactive species (Chaudhary et al. 2023). By enhancing the activity of these enzymes, silymarin reinforces the cellular antioxidant defense system, thereby reducing the overall oxidative burden on brain tissue during reperfusion.

Ischemia-reperfusion injuries (IRIs) by targeting carbon–carbon bonds of polyunsaturated fatty acids begin lipid peroxidation, a process in which ROS attack polyunsaturated fatty acids in cell membranes (Jurcau and Ardelean 2022). 

Nitrosative stress, involving NO formation, contributes to reacting with superoxide and producing ONOO^−^, which causes damage during I/R. Silymarin has been indicated to modulate the activity of NOS synthase and reduce nitrosative stress (Wang et al. 2022). Various studies indicated that by regulating NO levels, silymarin helps to balance beneficial signaling functions and the detrimental effects of excessive nitrosative stress (Chauhan et al. 2017; Gupta and Gupta 2017; Hou et al. 2010; Rathore et al. 2020; Surai 2015). 

### Mitochondrial protection

Silymarin's antioxidant effects protect mitochondrial function by preventing mitochondrial membrane permeabilization, maintaining the electron transport chain, and preventing mitochondrial dysfunction. By preserving mitochondrial integrity, silymarin contributes to the inhibition of mitochondrial dysfunction and the associated release of ROS (Esselun et al. 2019). During the ischemic phase, the brain has difficulty receiving sufficient oxygen and glucose from the blood, making it challenging to preserve the regular electrical activity of neurons. To cope with this stress, various brain cells work together. However, sudden blood reperfusion can lead to the accumulation of ROS and mitochondrial dysfunction (Brandes et al. 2014). The healthy function of mitochondria is essential to maintain a balance of ROS. Consequently, if mitochondria, the primary location of ATP synthesis and the cornerstone of redox homeostasis, are damaged, ATP reserves in the brain will be depleted, and oxidative stress levels will increase (Cheng and Pamenter 2021).

Raza et al. (Raza et al. 2011) indicated that silymarin administration at 200 mg/kg body weight dosage for 15 days to male Wistar rats with middle cerebral artery occluded, significantly protected the activity of Na^+^–K^+^ ATPase. Disruption of the mitochondrial membrane potential is a common consequence of I/R, which can lead to the opening of mitochondrial permeability transition pores (mPTP) and the trigger of pro-apoptotic factors and neurotoxic agents. Additionally, mitochondrial permeability transition pore opening has an important role in IRIs that releases cytochrome c, an apoptosis-inducing factor, and some procaspases (39). The administration of silibinin (200 µM) significantly inhibited ROS formation and mitochondrial membrane potential (MMP) decrease, which are indicators of mPTP opening in SH-SY5Y cells subjected to oxygen-glucose deprivation/re-oxygenation (Xie et al. 2014).

Silybin, a significant bioactive flavonolignan in silymarin, has been shown to preserve mitochondrial membrane potential, which is critical for maintaining the integrity and function of the mitochondria. By stabilizing the mitochondrial membrane potential and decreasing preserving brain-derived neurotrophic factor (BDNF) levels and pro-inflammatory cytokines, silybin prevents mPTP opening. It reduces the risk of such deleterious changes (Ramírez-Carreto et al. 2023).

Maintaining the integrity of mitochondria is crucial for proper electron transport chain function and ATP production (Cowan et al. 2019). During ischemia, the nuclear neurons may experience insufficient glucose storage and rapid depletion of ATP, potentially resulting in tissue necrosis. The mitochondria serve as the metabolic hub of ROS, playing a vital role in preventing such damage by ensuring a constant production and consumption of ROS. Additionally, these organelles generate most of the cell's required ATP (Mezhnina et al. 2022). During CI/RI, mitochondrial dysfunction is induced by an imbalance in ATP homeostasis and ROS accumulation. This leads to a further increase in ROS, exacerbating the disruption of ATP homeostasis and accelerating the onset and progression of CI/RI (Orellana-Urzúa et al. 2020).

Silymarin has beneficial effects on mitochondrial function by promoting the generation of new mitochondria, a process known as mitochondrial biogenesis. This can occur through upregulating peroxisome proliferator-activated receptor gamma coactivator 1-alpha (PGC1-α), a crucial regulator of mitochondrial biogenesis. By upregulating PGC-1α, silymarin can increase mitochondrial capacity to replace damaged mitochondria and maintain optimal function during recovery (Bin et al. 2017; Kheiripour et al. 2019). A study that was conducted by Li et al. reported that administering silibinin at concentrations of 5, 10, 20, 50, 100, or 200 Μm for 24 hours in hypoxia/reoxygenation-treated N2a cells inhibited mitochondria dysfunction by upregulated uncoupling protein 2 (UCP2), nuclear respiratory factor 1 (NRF1), and PGC1-α (Li et al. 2023).

### Anti-inflammatory and immunomodulatory effects

The neuroprotective properties of silymarin and silibinin can be attributed to their anti-inflammatory effects on CI/RI. When CI/RI occurs, it triggers an inflammatory response that can worsen tissue damage and neuronal injury. However, silymarin can help to mitigate this response by exerting its anti-inflammatory mechanisms (Khazaei et al. 2022). Several studies demonstrate that silymarin can inhibit the production and release of proinflammatory cytokines, including tumor necrosis factor-alpha (TNF-α), IL-1β, IL-6, IL-8, IL-10, IL-17, IL-23, carbon tetrachloride (CCL4), C-X-C motif chemokine 10 (CXCL10), C-reactive protein (CRP), and transforming growth factor-β1 (TGF-β1) serum levels (Darvishi-Khezri et al. 2021; Shahidi et al. 2017; Surai et al. 2024). Silymarin also can inhibit the expression of cyclooxygenase-2 (COX-2), NO production, inducible nitric oxide synthase (iNOS), prostaglandin (PG)-E2, and intercellular adhesion molecule (ICAM-1) expression (Chen et al. 2017). Hou et al. in their study, reported that 5 or 10 µg/kg administration of silymarin in a CI/RI model in rats can reduce iNOS, COX-2, and myeloperoxidase (MPO) expression and inhibit transcription factors, including nuclear factor-kappa B (NF-κB), STAT1 and IκB-α degradation. It also attenuated TNF-α, IL-1β, and NO production (Hou et al. 2010). Another study conducted by Moghaddam et al. revealed that administration of silymarin-loaded chitosan nanoparticles (15 mg/kg orally) to Wistar rats with bilateral typical carotid artery occlusion, reduced IL-6 and TNF-α (Moghaddam et al. 2020). 

Silibinin and its diastereomers, silybin, were investigated for anti-inflammatory effects. Silibinin at 50 and 100 mg/kg concentrations and silybin at 75 and 150 mg/kg dosage revealed promising inhibition on NF-κB activity. The mRNA expression was also reduced in experimental rats (Guo et al. 2014; Wang et al. 2012). 

Silibinin can also suppress the expression of adhesion molecules such as ICAM-1 and VCAM-1 which facilitate the recruitment of immune cells to the injury site. By doing so, silibinin reduces inflammation and prevents secondary damage (Sun et al. 2023).

In a study, silibinin was observed to reduce lipopolysaccharide (LPS)-induced oxidative-nitrosative stress in C6 astrocytoma cells effectively. The study also revealed that LPS significantly inhibits astrocytes' activity. Furthermore, molecular modeling studies suggest that silibinin binds to the binding sites of critical downstream cascades such as p38 MAPK, CX3CR1, and P2X4, which are involved in glial cell activation and neuroinflammation (Fernandes et al. 2018).

The initial prominent characteristic of the neuroinflammatory triangle during a hypoxic pathophysiological state is the compromised integrity of the BBB. This is when astrocytes located in the penumbra, pericytes, and BMECs start to release ET-1, leading to an imbalance of endothelin-1 (ET-1) and NO (Shaheryar et al. 2021). This situation leads to overexpression of adhesion molecules such as ICAM-1, VCAM-1, and ELAM-1 in or on brain microvascular endothelial cells (BMECs). These adhesion molecules promote the movement of leukocytes across the endothelium layer.

Additionally, ET-1, a peptide secreted by endothelial cells, has the capacity to enhance the expression and release of matrix metalloproteinases (MMPs) from brain microvascular endothelial cells (BMECs). These MMP enzymes can degrade inter-endothelial junctional proteins such as Claudin-1, Claudin-5, Occludin, Zonula occludens-1 (ZO-1), and junctional adhesion molecule-A (JAM-A), thereby facilitating the trans-endothelial migration of leukocytes (Shaheryar et al. 2021; Williams and Luscinskas 2011). The integrity of the barrier, normally tightly regulated by BMECs, is disrupted, leading to inflammatory cytokine release, systemic immune cell infiltration, and fluid leakage, which causes brain edema. This cytokine release also activates meningeal mast cells, exacerbating BBB damage and resulting in prolonged ischemia of already deprived brain tissue (Shaheryar et al. 2021). 

Silymarin effectively preserves BBB integrity, preventing the uncontrolled entry of inflammatory cells. It is vital to maintain BBB integrity as it helps to minimize the inflammatory response and reduces the risk of further damage to the brain tissue (Ranjan and Gautam 2023). Research has shown that when silymarin is used in conjunction with a P-glycoprotein (P-gp) substrate drug, the distribution of the drug across the BBB is enhanced. P-gp is a transporter protein typically present in the BBB and functions by blocking substrate drugs from entering the brain (Ravikumar Reddy et al. 2016).

### Anti-apoptotic effects

During an ischemic event, an increase in the ROS formation occurs, leading to intrinsic apoptotic signaling and neuronal death (Mao et al. 2022). This occurs because the mitochondria in neurons absorb extracellular calcium ions (Ca^2+)^ due to an increased respiratory burst, leading to calcium accumulation. After these molecular processes, the proteins responsible for preventing cell apoptosis (BCL-2 and BCL-x) in the mitochondria lose their function. As a result, the permeability of both the inner and outer mitochondrial membranes increases (Shaheryar et al. 2021). This facilitates the entry of Bax proteins from the cytoplasm into the mitochondria, where they associate with Bar proteins to form tunnel-like structures in the outer mitochondrial membrane, creating Bax-Bar pores. These pores allow the release of pro-apoptotic proteins such as AIF, Cy A, APAF-1, and cytochrome c, which subsequently activate caspase 8 and initiate apoptosis (Shaheryar et al. 2021). 

On the other hand, when the lipids are affected by oxidative damage during I/R, it can lead to several adverse effects such as cell membrane breakdown and mitochondria swelling (Kuznetsov et al. 2019). This swelling is caused by opening a pore in the mitochondria known as mPTP, which is triggered by proteins reacting with other proteins and ROS. Once the mPTP is open, it activates P53 which can also react with other proteins in the Bcl-2 family to release cytochrome c from the mitochondria (Rottenberg and Hoek 2021). This then initiates a series of events, leading to apoptosis or cell death. ROS can also activate two different pathways, the c-Jun N-terminal kinase (JNK) and p38 MAPK pathways, both of which are activated by a protein called apoptosis signal-regulating kinase 1 (ASK1), which ultimately leads to apoptosis (Sun et al. 2018).

Silymarin has been proven to suppress the activation of caspases which are the executioners of apoptosis. Caspases, especially caspase-3, play a central role in cleavage cellular proteins and dismantling cellular structures during apoptosis (Parrish et al. 2013). The Bcl-2 family of proteins regulates the intrinsic (mitochondrial) apoptotic pathway (Hardwick and Soane 2013). Silymarin has been reported to modulate the expression of Bcl-2 family members, including increasing the anti-apoptotic protein Bcl-2 and decreasing pro-apoptotic proteins such as Bax. This modulation of Bcl-2 family proteins promotes the inhibition of mPTP opening, releasing cytochrome c and subsequently preventing the initiation of the apoptotic cascade. In their study, Raza et al. (Raza et al. 2011) indicated that silymarin (200 mg/kg body weight for 15 days) in male Wistar rats with middle cerebral artery occluded, reduced p53, and caspase-3 and -9 expressions. Silibinin at various concentrations was investigated for its effects on CI/RI in rat models and oxygen-glucose deprivation. These studies revealed that 1–200 µM and 200 mg/kg of silibinin can reduce apoptosis by AMPK signaling activation. It also reduces caspase-3 activity and apoptosis. Under oxidative stress, it can prevent cell apoptosis by increasing procaspase-3 levels, balancing Bcl-2, and inhibiting Bax expression. Additionally, it can prevent autophagy by reducing LC3-II and Beclin-1 levels (Li et al. 2023; Wang et al. 2016; Xie et al. 2014). Moreover, Rathore et al. and Mokhtari et al. reported in their study that silymarin loaded with nanoparticles can potentially reduce caspase-3 expression and increase Bcl-2 expression. Bioavailability was increased by nano-silymarin treatment (Mokhtari Sangdehi et al. 2022; Rathore et al. 2020).

### Improve histopathological changes and decrease cerebral infarct size

The first indication of brain cell damage is the contraction or expansion of neurons. This can lead to "red neurons" forming due to eosinophilia, nuclear pyroptosis, and other cytoplasm-related necrotic changes. Additionally, there may be signs of swelling, such as empty spaces within the tissue and enlarged spaces between cells and blood vessels (Vera et al. 2012). This condition is characterized by a patchy area of discoloration where the grey and white matter of the brain becomes blurred. This can result in the loss of neurons and gliosis over time. Unlike other organs affected by ischemia, the brain undergoes liquefactive necrosis, resulting in a viscous substance with inflammatory cells and cellular debris, including neutrophils (Vera et al. 2012).

 Several studies have shown that silymarin administration to Wistar rats with CI/RI is supposed to improve water content, attenuate edema, and decrease CI/RI brain infraction size (Chauhan et al. 2017; Hou et al. 2010; Moghaddam et al. 2020; Muley et al. 2013; Muley et al. 2012). Hypoxic changes, vacuolar changes, congestion, and mild degree of phagocytosis, edema of neurons were evaluated as pathological changes in induced CI/RI rats. The results revealed that 100 and 200 mg/kg of silymarin could improve the histopathology of the brain (Muley et al. 2012). Karabag et al. reported that 200 mg/kg silymarin was given as ip 5 min before the tourniquet (ischemia), which resulted in reduced histopathologic damage scores in the studied animals (Karabag and Koçarslan 2020). Histopathological alterations in hematoxylin and eosin-stained coronal brain sections of middle cerebral artery occlusion (MCAO) rats were reversed following 7 days of administration of silymarin encapsulated in nanoparticles (Rathore et al. 2020). 

### Improve neurobehavioral tests

Patients with ischemic stroke may experience neuropsychiatric complications such as aggressive behaviors, depression, anxiety, and mood changes. The more severe the ischemia, the more severe the behavioral changes in the post-stroke period. Behavioral symptoms positively correlated with more functional impairment (Kim 2016; Rush et al. 2010). 

Moghaddam et al. revealed that administering silymarin-loaded chitosan nanoparticles (15 mg/kg orally) to Wistar rats with bilateral typical carotid artery occlusion reduced depressive-like behaviors (Moghaddam et al. 2020). Behavioral changes, including memory impairment, motor control, and neurological scores, were investigated in the Muley et al. study (Muley et al. 2012). Their study showed that silymarin at 200 mg/kg dosage improved behavioral scores in rats with focal I/R. Another study evaluated behavioral changes by assessing the effects of silymarin on transfer latency and fall latency in Wistar rats with bilateral typical carotid artery occlusion. The results improved water content, and histopathological alterations (Muley et al. 2013). Rathore et al. evaluated the effects of silymarin encapsulated inside collagen-based polymeric nanoparticles' effects on the rota-rod test, (for assess motor activity), flexion test and grip strength test (behavioral tests). They demonstrated that nano-silymarin in 100 µg/kg b.wt for 7 days can improve behavioral changes due to CI/RI (Rathore et al. 2020). Furthermore, silibinin (200 mg/kg) has promising effects on neurological score and locomotor activity in male Wistar rats with middle cerebral artery occlusion-induced focal cerebral ischemia (Chauhan et al. 2017). Rats with global CI/RI were given silymarin 14 days before to bilateral typical carotid artery occlusion, and depressive-like behaviors were improved after intervention (Moghaddam et al. 2020).

### Cell signaling pathway

By modulating the Nrf2/ARE pathway (Nuclear factor erythroid 2–related factor 2 / Antioxidant Response Element), silymarin helps produce detoxifying and antioxidant enzymes (Vargas-Mendoza et al. 2020). Nrf2 is a transcription factor that, when activated, moves to the nucleus and binds to ARE, producing antioxidant enzymes that safeguard against oxidative damage (Ngo and Duennwald 2022). When a tissue is exposed to reactive oxygen species (ROS), the body triggers a complex called Nrf2/ARE, which causes Nrf2 to separate from the complex and move to the nucleus. Once there, Nrf2 activates specific genes containing a sequence called an antioxidant response element (ARE) in their promoters. This activation produces endogenous antioxidants, including glutaredoxins (GRx), SOD, glutathione (GSH), and CAT. These antioxidants help the body defend against oxidative stress (Jurcau and Ardelean 2022). Li et al. in their study reported that 5, 10, 20, 50, 100, or 200 Μm of silibinin for 24 h administration in Hypoxia/reoxygenation-treated N2a cells upregulated Nrf2, HO-1, NAD(P)H: quinone oxidoreductase 1 (NQO1) in rats with models (Li et al. 2023). 

Silymarin demonstrates its anti-inflammatory properties by inhibiting the NF-κB pathway, a key controller of inflammatory reactions. Activation of NF-κB induces the production of pro-inflammatory cytokines (IL-4, IL-10, IL-13, TGF-β) and adhesion molecules (IL-6, IL-1β and ICAM-1) (Surai et al. 2024), thus intensifying tissue injury in cerebral ischemia-reperfusion. A study reported that Silymarin encapsulated inside collagen-based polymeric nanoparticles at 10 µg/kg b.wt concentration in albino Wistar rats with middle cerebral artery occlusion attenuated expression of NF-κB, iNOS (Rathore et al. 2020), silymarin has been reported to modulate the NF-κB signaling pathway, which is responsible for the upregulation of various inflammatory mediators. NF-κB activation leads to the transcription of pro-inflammatory genes. Moreover, silymarin also suppresses the NF-κB signaling pathway (involved in cytokine production) and NLRP1/NLRP3 inflammasomes (Matias et al. 2019).

Silymarin has been found to modulate cell death pathways through its regulation of the PI3K/Akt pathway (Phosphoinositide 3-kinase/Protein Kinase B). Silymarin modulates various signaling pathways associated with neurotrophic effects, including the MAPK and PI3K/Akt pathways, which are involved in cell survival, growth, and differentiation. By modulating these pathways, Silymarin contributes to the observed neurotrophic effects in the context of CI/RI (Sun et al. 2018). Silymarin has been shown to activate survival pathways such as the phosphoinositide 3-kinase (PI3K)/Akt pathway. Activation of Akt improves cell survival by inhibiting pro-apoptotic factors and modulating downstream effectors involved in apoptosis. By activating these survival pathways, Silymarin protects against apoptotic cell death in the reperfused brain tissue (Guo et al. 2014).

### Other possible protective mechanisms

Neurotrophic effects and anti-excitotoxicity may be involved in silymarin's neuroprotective effects against CI/RI (Borah et al. 2013). BDNF is an essential element that supports the development and endurance of neurons via exosomes from BDNF-overexpressing HEK293 cells and preventing apoptosis in rats with CI/RI (Wang et al. 2021). Studies have revealed that silymarin can enhance the expression of BDNF in the brain. This discovery is noteworthy as BDNF plays a pivotal role in numerous processes, such as preserving neurons, encouraging neuronal flexibility, and triggering neurogenesis (generating new neurons from neural stem cells) (Ranjan and Gautam 2023; Thakare et al. 2017; Yan et al. 2015).

Studies have found that silymarin can stimulate neurogenesis in the brain's hippocampus region, which plays a vital role in learning and memory. By promoting the formation of new neurons, silymarin could help to replenish neuronal populations that may have been affected by ischemia-reperfusion, ultimately aiding in the recovery of cognitive function (Yan et al. 2015).

Excitotoxicity is closely associated with generating ROS and oxidative stress. Moreover, silibinin may protect against excitotoxicity, characterized by excessive glutamate release and subsequent neuronal damage (Kim et al. 2017). Silymarin helps regulate glutamate release, which helps preserve a balance in neurotransmission and prevents the harmful effects associated with excessive glutamate levels (Lu et al. 2020). Excitotoxicity is often linked to an influx of Ca^2+^ ions into neurons through the overactivation of N-Methyl-D-aspartate (NMDA) receptors, resulting in cellular damage during IRI. Silymarin has been shown to reduce intracellular Ca^2+^ overload by potentially modulating Ca^2+^ channels or preventing excessive Ca^2+^ entry through glutamate receptors. By regulating intracellular Ca^2+^ levels, silymarin protects neurons from the detrimental effects of calcium-induced excitotoxicity (Tan et al. 2015).

**Figure 3:**
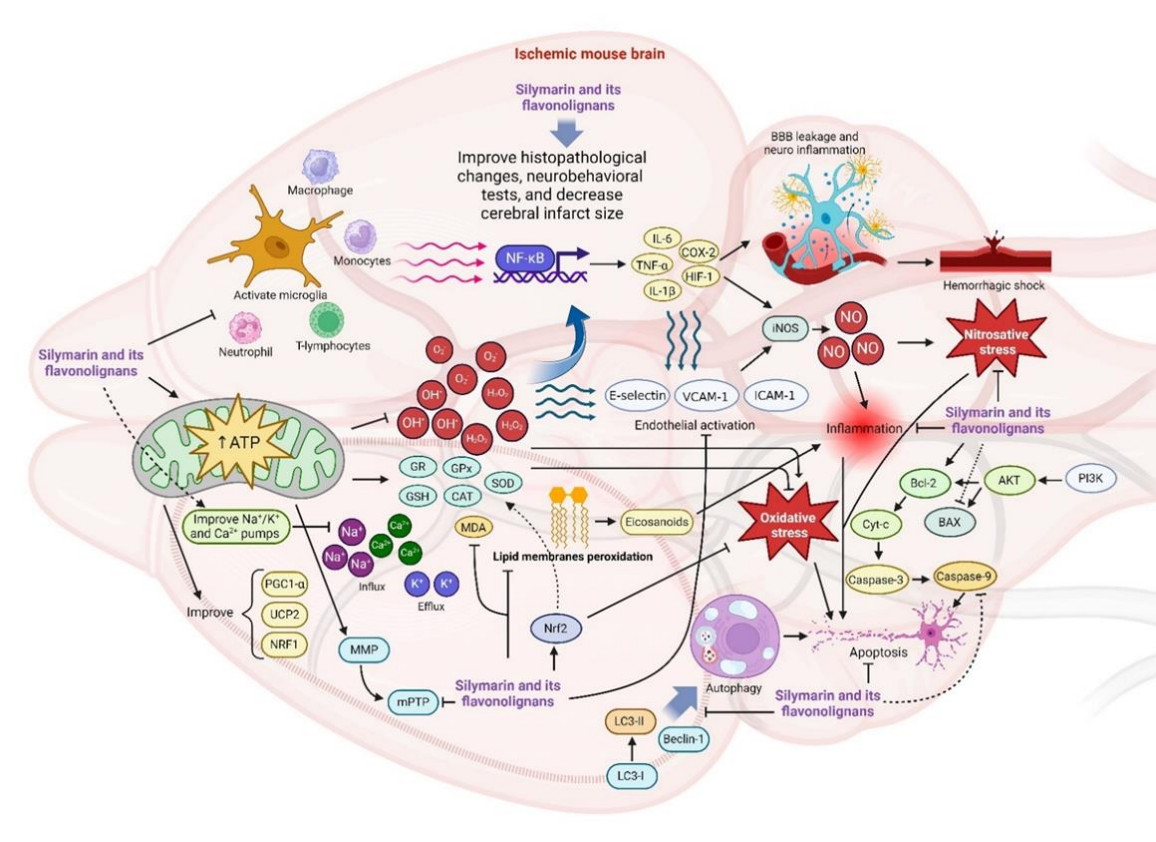
Possible silymarin and silibinin protective mechanisms against CI/RI through improved endogenous antioxidants (GSH, CAT, and SOD) and reduced lipid peroxidation, NO, and MDA levels. It also enhances the Nrf2 expression and upregulated HO-1 and NQO1. They also protected mitochondrial function by improving the activity of Na^+^–K^+^ ATPase, activating mPTP. Moreover, upregulated PGC1-α, UCP2, and NRF1, and reduced iNOS, COX-2, and MPO expression. They inhibited NF-κB, IκB-α degradation, TNF-α, IL-1β and IL-6. Anti-apoptotic properties were revealed by increasing protein Bcl-2 and reducing p53, Bax, caspase-3, and 9 expressions. (The figure Created in: biorender.com)

Overall, the main mechanisms of silymarin and silibinin are illustrated in Figure 3.

### Side effects

While the studies reviewed did not reveal any severe side effects or cytotoxicity, it is important to note that oral milk thistle administration may cause gastrointestinal issues such as diarrhea, nausea, abdominal bloating, dyspepsia, flatulence, stomach pain, anorexia, and changes in bowel habits, as well as headaches, skin reactions (including rashes and itching), neuropsychological disturbances, arthralgia, rhinoconjunctivitis, and allergic reactions (La Vecchia 2001). It is essential to remember that the included studies were conducted using *in vivo* and *in vitro* designs. At concentrations of 100 μM, silybin, silydianin, and silychristin are non-toxic to cells and DNA. When consumed at therapeutic levels, silymarin is considered safe for human use. Even at high doses of 700 mg taken three times daily for up to 24 weeks, it is generally well-tolerated. However, specific individuals may experience gastrointestinal issues like nausea and diarrhea (Soleimani et al. 2019). Silymarin is safe for therapeutic use in humans, even at high doses of up to 700 mg taken thrice daily for 168 days. This is good news for those relying on silymarin to support liver function and other health benefits. However, it is worth noting that some individuals may experience mild gastrointestinal discomforts such as nausea and diarrhea (Ranjan and Gautam 2023). The liver relies on the activity of specific cytochrome enzymes, namely CYP3A4 and CYP2C9, to metabolize some medications. Research has shown that silymarin may hinder the liver's ability to metabolize certain medications properly by inhibiting these crucial cytochrome enzymes (Doehmer et al. 2008; Faisal et al. 2021; Jancová et al. 2007; Kawaguchi-Suzuki et al. 2014; Zhang et al. 2023). 

Additionally, the pharmaceutical utilization of silymarin encounters further obstacles as more research studies on its drug interactions are necessary (Soleimani et al. 2019). Administration of silymarin for extended periods at doses up to 400 mg per 1 kg did not impact fish blood's clinical and biochemical parameters. Nonetheless, when fish were orally given Silymarin at 800 mg/kg, it resulted in cytotoxicity and alterations in the biochemical blood parameters (Banaee et al. 2011). 

### Limitations

The included studies have used different experimental designs and methodologies, leading to heterogeneity in I/R animal/cell models, dosage regimens, and treatment duration, which could make it challenging to synthesize and generalize the findings. Furthermore, preclinical studies dominate the available literature, and their translational relevance to humans may need to be fully understood. Well-designed clinical trials are needed to establish a more robust understanding of the therapeutic potential of silymarin in CI/RI. Moreover, the effective delivery of silymarin is challenging due to the limited permeability of the BBB. Studies have revealed that P-gp, an efflux transporter, prevents several therapeutic agents, including Silymarin, from entering the brain through the BBB (Ravikumar Reddy et al. 2016). 

*In vivo* and *in vitro* studies revealed promising biological effects of silymarin and silibinin on CI/RI. The reviewed studies offer valuable insights into the potential neuroprotective properties of Silymarin in mitigating the impact of ischemia-reperfusion events. The evidence synthesized from diverse experimental models suggests that silymarin may have beneficial effects, including anti-inflammatory antioxidant antiapoptosis mechanisms, modulation of histopathological changes, decreased cerebral infarct size, and improvement in neurobehavioral tests. However, in-depth clinical trials are needed to draw more accurate and reliable results in this field. 
